# A bioink by any other name: terms, concepts and constructions related to 3D bioprinting

**DOI:** 10.4155/fsoa-2016-0044

**Published:** 2016-07-22

**Authors:** William G Whitford, James B Hoying

**Affiliations:** 1 GE Healthcare Life Sciences, 925 West 1800 South, Logan, UT 84321, USA; 2 Advanced Solutions Life Sciences, 1901 Nelson Miller Parkway, Louisville, KY 40223, USA

**Keywords:** 3D printing, 4D printing, biofabrication, bioinks, biomanufacturing, bioprinting

Recently, specialized applications of 3D printing have extended into the biological realm. As the field is so new, related activities are being approached from groups with disparate specialties and backgrounds. Therefore, few firm, unambiguous and universally understood definitions have been established. Many have local and connotative meanings, and most have both unique and overlapping aspects to their evolving applications. Also, questions arise regarding the distinction between some of the almost homonymous terms – and even such confusion as to the meaning of the prefix ‘bio’ in terms. For example, some may think the difference between 3D biomaterial printing and 3D printing is a biological nature and/or biocompatibility of materials in the printed object. While for others, the difference reflects a biomedical application of the object post-printing. Our intent is to introduce common usages of 3D bioprinting-related terms in the context.

## 3D printing

3D printing is now a well-established technology and rapidly gaining utility in industry [1,2]. As an implementation of ‘additive manufacturing’ and ‘direct digital manufacturing’ it refers to a particular means of product fabrication; specifically, processes where successive layers or rows of material are deposited under computer control to directly create a 3D object. There are a variety of mechanisms for this controlled deposition fabricating the object directly or via a printed mold from which the final product is cast.

## 4D printing

In 4D printing, the fourth, or extra, dimension is typically time-dependent. Products here are a special case of ‘self-actuating materials’ [3–5] providing a smart, environmentally responsive change in structure or functionality that is engineered and self-actuated. Thus, an intrinsic feature of a 3D-printed material leads to subsequent, progressive changes in, for example, the shape or topology of the printed object. Careful definition of the post-printing change is required, as for example, dropping a printed object on the floor may cause a change in its shape, but it is neither designed nor self-originated. These changes in shape or size or functionality are usually influenced by such inputs as heat, light, humidity or air pressure.

## Medical 3D printing

One exciting example of this biomedical application of 3D printing is 3D printed pills – a new dosage form in late stages of development [6]. 3D printed pills provide many advantages, from improved chemical stability, pill dissolution and medication adsorption to multiple unit-dosing of disparate medications. They also promise to support pills as easier to take and allow here-to-for impossible pill design. They may even be customizable – supporting personalized medicines and therefore more inexpensive, accurate and effective dosing. An example of ambiguity in terms is that many refer to the action of 3D printing parts for prosthetic implants as 3D biomaterial printing or biofabrication and as an implementation of medical 3D printing*.* Regulatory designations beyond the scope of this paper include 3D printing toward active implantable medical devices, nonimplantable medical devices or *in vitro* diagnostic medical devices. Medical 3D printing has also been used to refer to patient-specific anatomical models, often derived from patient-specific image data sets, used to guide surgical strategies. Technically, no living or biological components are used in printing of a model; typically resins or thermoplastics are used. However, the medical-related application lends itself to the terms used.

## 3D biomaterial printing & bioadditive manufacturing

The generation of complex 3D biomedical devices and enhanced scaffolds has driven the need for 3D printing with biocompatible (or ‘biofunctionalizable’) materials such as natural and synthetic polymers, polymerizable fluids, ceramics and metals. In this (most common) usage, it is the product’s compatibility with cells, tissues and humoral systems that drives the ‘bio’ component of the term. ‘Biocompatible implants’, ‘biopapers’ and other scaffolds require appropriate macro-, micro- and nano-level properties. While cells are not typically a component of such printed devices, biocompatibility is required to support subsequent cell-interaction. Examples of these properties include micro-architecture supporting cell adhesion and matrix integration, as well as heterogeneous multifold scaffolds providing appropriate release kinetics of loaded biomolecules enabling synchronization of regenerating tissue [7–9]. In biomaterial applications some refer to any method of solid free form fabrication toward a biological application as 3D printing, while others reserve it for liquid binder-based inkjet technology [10,11]. Related expressions in this arena include ‘additive biomanufacturing’ and ‘bio-medical additive manufacturing’. The 3D biomaterial printing and bioadditive manufacturing terms are employed by some as 3D bioprinting [12–14].

## Biofabrication & biomanufacturing

The general objective is the production of complex biological products from such raw materials such as (in)organic molecules, extracellular matrices, biomaterials and living elements (i.e., cells) [15,16]. Historically, manufacturing refers to the formation of a complete, usable product while fabrication efforts lead to the formation of a part or sub-assembly used in a more comprehensive manufacturing program. For example, a company purchases many fabricated parts that are then assembled into a manufactured car. With these traditional definitions in mind, 3D printing of a biomaterial scaffold would be referred to as biofabrication while integration of this scaffold with cells and/or other components would constitute biomanufacturing of the final product. With living systems, these classical definitions may be blurred as many biological ‘parts’ are contained, functional products. For example, a biological ‘subassembly’, such as a prevascularized matrix, intended to be used in manufacturing a larger, more complex and complete tissue product can itself be used as is. However, biomanufacturing may also refer to processes in which biological systems manufacture a product, such as operation of a cultured cell system to secrete biomolecular products, an activity traditionally termed biosynthesis [17]. Regardless, practically speaking, technologies employed to originate a biological structure, such as molding, casting, stereolithography, cell seeding and 3D bioprinting reflect fabrication approaches. Meanwhile, approaches enabling the integration of these fabricated structures into or production of a product (e.g., pick-and-place, systems engineering) reflect more manufacturing approaches. It is likely these terms will become more defined as the field evolves [17]. Reflecting this broader evolution, new 3D bioprinter designs have emerged using a manufacturing robot as the core fabricating technology [18].

## Bioprinting, 2D bioprinting & 3D bioprinting

All three terms refer to biofabrication through the deposition of micro-channels or -droplets of living cells with or without additional structural materials. The most common term, 3D bioprinting (3DBP), describes the fabrication of 3D, engineered living (often cell-based) models, tissues and organs. These are being used for drug discovery, pharmaceutical and environmental toxicology assays, *in vitro* models of organism development and disease, and production of engineered human tissues and organs [5,19]. In 3DBP, the cell-laden fluids or bioinks are built upon each other and become any number of very small or rather large biological structures. 2D bioprinting employs related equipment to organize cells in monolayers for such applications as cell-based assays and models [20].

The actual mechanics of this deposition of cells and matrix vary greatly between bioprinters and bioprinting applications. But regardless of the printing specifics, these printed objects composed of cells, biopolymer hydrogels or synthetic matrix materials present revolutionary promise in research, diagnostics and therapeutics [9]. Many are working on standardized 3D human tissues for predictive toxicology and preclinical testing because 3DBP tissues recapitulate many aspects of *in vivo* tissue architecture and function. It is believed they will provide many distinct advantages over nonhuman animal models. The most common bioprinting, ‘scaffold-based bioprinting’, involves fluids composed of cells, nutrients and (biomedical) matrix materials*.* ‘Scaffold-free bioprinting’ [21] involves the manipulation of concentrated cells and their own extracellular matrix in a ways that exogenous natural or synthetic matrix materials are not required for immediate structural integrity.

## 4D bioprinting

As 3DBP is so new, we might expect that a further development, 4D bioprinting (4DBP) would be in a stage of definition and development. As in 4D printing, we are here concerned with programmed, anticipated and self-actuated changes occurring well after the printing operation (Box 1) [3,22].

One example of 4DBP is producing an assembly of micro-droplets of cells in the general shape of final structure, designed such that they will eventually coalesce and shape-morph into the final intended structure. Some limit the change, as in 4D printing, to the construct’s size, shape or organization. Others suggest that the self-actuated changes in 4D bioprinting might allow developments other than shape-morphing. These other developments include cellular differentiation, polarization and tissue patterning as well as matrix evolution or functionality development – all of which reflect the robust adaptability intrinsic to most biological systems [23]. Importantly, from a fabrication perspective, 4D bioprinting will undoubtedly entail strategies for constraining constructed environments in such a way to direct the 4D/emergent behavior/activity/phenotype to a desired outcome (Box 2).

## Biomimetic 4D printing

Related to both 4D printing and 4DBP, biomimetic 4D printing involves predicted shape-morphing of composite-material printed objects following the dynamic architectures of living organisms. One example of this is 3D morphology development through post-printing changes in the printed objects hydration. In this case, the engineers have programmed lamellar hydrogel-printed structures with restricted, asymmetrical swelling. This controlled directional swelling of specifically aligned parts of the printed object determines a final shape after the object absorbs water [24]. The difference between biomimetic 4D printing and simple 4D printing appears to be the mechanism of 4D change. In this particular case, the anisotropic hydration initiated warping of the printed product, mimicking that in a plant.

## Bioinks

The fluids that 3D bioprinters deposit have been referred to as bioprinting inks or bioinks [25] and are corollary to biopaper. They are basically a fluid containing nutrients and/or matrix components and/or cells. In fact, a critical step in the bioprinting process involves selection or design of this bioink, as its composition is based upon the type of printing employed, and the sequence of product construction (Box 3). Several (biomedical) matrix materials have been specified for scaffold-based bioprinting [26,27]. They range from hydrogels built from such materials as alginate, collagen, fibrin, gelatin methacrylate, hyaluronic acid and block co-polymers to microcarriers or decellularized matrices. Some printing techniques present a strong reliance upon the nutrient and factor components of the bioink.

One ambiguous aspect of the term is that some use it (or variations) to refer to a cell-free material. Some condition the term for applications which may or may not contain living cells as ‘cell-encapsulating inks’ or ‘acellular inks’ [8]. Others do not. A matrix-containing fluid employed in a bioprinting process to simply generate an empty space or separate other active elements of the object can be referred to as a ‘sacrificial bioink’ [26].

## Conclusion

Many activities related to the 3D printing of a variety of materials for biotechnical and biomedical applications are now in development or actual use [28]. Many of the terms employed to identify them and their materials are in a stage of refinement. From the manufacturing of devices to synthetic polymers destined for biomedical implantation (biofunctionalizable 3D biomaterials) to complex fluids supporting living cells during synthetic tissue construction (a bioink), we find specific terms identifying a number of distinct materials designed for very different 3D bioprinting processes.

**Box 1.**  **4D bioprinting characteristics.**

Smart, environmentally responsive biological structure
Composed of various cells and biocompatible matrices
Undergoing designed and self-originated development
Responding post-printing to an environmental change

**Box 2.**  **Proposed types of 4D bioprinting.**

**Shape change:** a smart biopolymer and cells which changes its 3D configuration (or shape) upon stimulation
**Size change:** an *in vivo* printed cell device (e.g., hydrogel) is implanted in the body, then biological activities leave tissue as its absorbed
**Pattern change:** cell micro-droplets (± exogenous matrix) printed in a particular pattern, then stimulated to a pre-envisioned new pattern
**Phenotype change:** pre-engineered changes in nonstructural but biologically-relevant characteristics, such as the cellular assembly’s CD marker type or cell polarization

**Box 3.**  **Bioink design properties.**

Cell-specific formulation needs
Heightened pH buffering demand
Addressing component 3D gradients
Specifically control or inhibit apoptosis
Support or inhibit further differentiation
Printer-determined hydrodynamic stress
Co-culturing and tissue environment effects
Serum- and xeno-free and protein-free ideal
Address altered cell metabolism rates and flux
High plastic mass-to-medium volume ratio effects
Unique matrix and matrix-active component effects
Active and passive rheology requirements and effects
